# Pathogenesis and virulence of *Candida albicans*

**DOI:** 10.1080/21505594.2021.2019950

**Published:** 2021-12-29

**Authors:** José Pedro Lopes, Michail S. Lionakis

**Affiliations:** From the Fungal Pathogenesis Section, Laboratory of Clinical Immunology and Microbiology (LCIM), National Institute of Allergy and Infectious Diseases (NIAID), Bethesda, MD, USA

**Keywords:** *Candida albicans*, candidiasis, pathogenesis, virulence, immunity, host-pathogen interactions

## Abstract

*Candida albicans* is a commensal yeast fungus of the human oral, gastrointestinal, and genital mucosal surfaces, and skin. Antibiotic-induced dysbiosis, iatrogenic immunosuppression, and/or medical interventions that impair the integrity of the mucocutaneous barrier and/or perturb protective host defense mechanisms enable *C. albicans* to become an opportunistic pathogen and cause debilitating mucocutaneous disease and/or life-threatening systemic infections. In this review, we synthesize our current knowledge of the tissue-specific determinants of *C. albicans* pathogenicity and host immune defense mechanisms.

## Introduction

As one of the largest eukaryotic kingdoms, fungi have a variety of life cycle patterns with adaptations in metabolism and morphogenesis that enable them to adjust to the changing ecosystems [1]. Despite being estimated to have between 1.5 and 5 million fungal species, only a few hundred of those can cause clinical disease in humans [2]. The phylum Ascomycota contains some of the most successful human pathogens; these include virulent fungi that can infect individuals without immune compromise such as *Histoplasma, Blastomyces, Coccidioides*, and *Paracoccidioides* species as well as opportunistic fungi that cause disease primarily in immunosuppressed individuals such as *Aspergillus, Fusarium, Scedosporium*, and *Candida* species.

**Figure 1.**
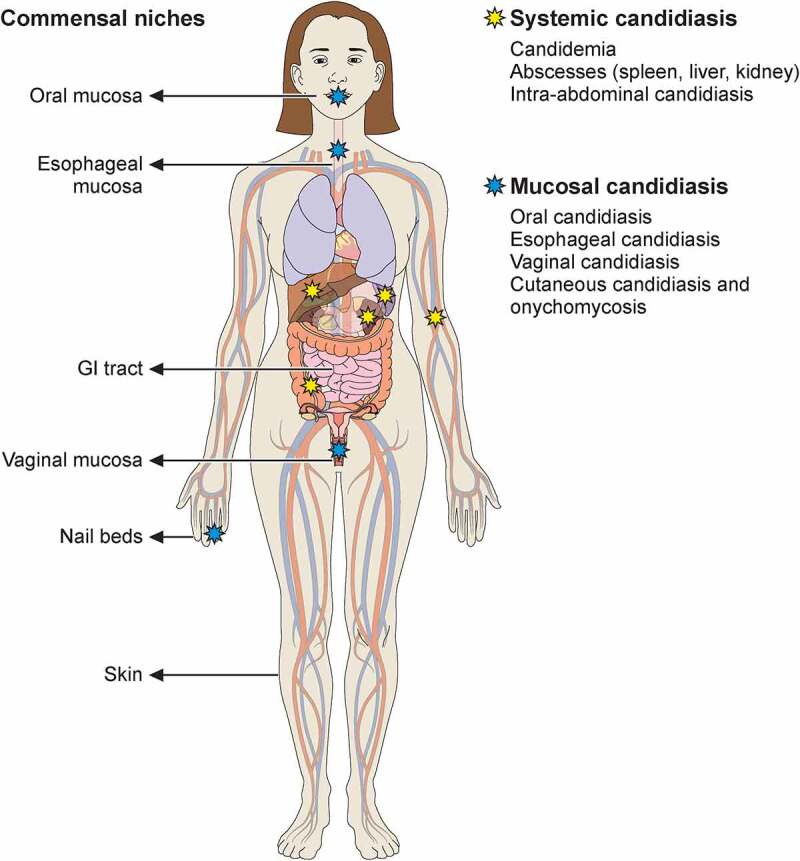
Commensal sites of *C. albicans* in the human body and clinical manifestations of *C. albicans* infection. Taking advantage of its commensal niches in the oral and genital mucosal surfaces and gastrointestinal tract, *C. albicans* can cause invasive disease (yellow star) and mucosal disease (blue star) in several tissues. Illustration created with BioRender.com.

*Candida* species are responsible for the majority of human infections caused by fungal pathogens. Members of these species include the most frequent cause of opportunistic infections, *Candida albicans*, the drug-resistant *Candida glabrata*, the new global public health threat *Candida auris*, and other emerging species such as *Candida tropicalis, Candida parapsilosis*, and *Candida krusei* [3,4]. In this review, we focus on *C. albicans* and the reader is referred to excellent overviews of non-*albicans Candida* species elsewhere [4–8]. Existing as a commensal in a large proportion of the human population, *C. albicans* colonizes the oral, gastrointestinal, and genital tracts asymptomatically [9,10]. However, upon perturbation of barrier integrity and/or host immune responses, the fungus can migrate through the epithelium and access deep-seated anatomical niches to cause infection. Medically important infections caused by *C. albicans* can broadly be classified into two subtypes: mucosal and systemic (Figure 1). Mucocutaneous surfaces primarily affected by *C. albicans* are the vaginal (vulvovaginal candidiasis [VVC]), the oral (oropharyngeal candidiasis [OPC]), the esophageal (esophageal candidiasis [EPC]) and, less often, the nails (onychomycosis). Skin candidiasis is exceedingly uncommon and may rarely occur in a small proportion of patients with certain inborn errors of immunity (see below). Mucosal candidiasis, particularly in the form of VVC, can occur in people with intact immune functions, although immunocompromised individuals are at higher risk for increased frequency, severity and/or recurrence of mucosal infections. Systemic candidiasis affects sterile body sites such as the bloodstream and can involve the central nervous system (CNS), liver, spleen, heart, and/or kidneys. It can also involve the intra-abdominal compartment with or without bloodstream spread [4]. Systemic candidiasis is associated with high mortality despite the administration of antifungal therapy [4].

To effectively cause mucosal and/or systemic disease and withstand the subsequent antifungal host response and antifungal drug treatment, *C. albicans* employs several virulence traits principal of which are: a) temperature adaptation; b) adhesion and invasion; c) nutrient acquisition; d) immune evasion; and e) drug tolerance. In this review, we will focus on *C. albicans* pathogenicity related to these five traits tracking the fungus route from being a mucosal commensal to becoming an opportunistic pathogen causing mucosal and/or systemic disease. We will also highlight the roles of the innate and adaptive immune systems and of the mucocutaneous barrier in preventing and curtailing disease and we will briefly discuss key antifungal drug targets and the countermeasures employed by *C. albicans* to achieve antifungal drug tolerance and resistance.

## *C. albicans*: From commensal to opportunistic pathogen

*C. albicans* is a ubiquitous fungal organism residing in the mucosa of humans while also living in certain environmental reservoirs [11]. Human colonization of the mouth, vagina, and gut typically develops during infancy, primarily during vaginal delivery or breastfeeding [12,13]. In mice, which are not naturally colonized by *C. albicans*, colonization resistance relies on the presence of intact endogenous microbiota [14] and antimicrobial peptides (AMP) such as CRAMP, a peptide related to the human AMP cathelicidin LL-37 [15,16].

Recently, it was shown that immune selection by intestinal IgA against *C. albicans* filaments suppresses harmful fungal effectors while improving the competitive fitness of *C. albicans* yeast as a commensal [17]. Within the gut, *C. albicans* helps shape the composition of the healthy microbiota by inhibiting multiple dominant genera of gut bacteria [18] and local inflammation [19]. The presence of *C. albicans* in the gut is linked to an increase in splenic IgG-producing B cells and systemic antifungal IgG conferring protection against candidemia [20–22]. In mice, multiple passages of *C. albicans* resulted in fungal adaptation toward colonization and improved protection against subsequent nonspecific infections in a lymphocyte-independent manner [23].

*C. albicans* carriage is asymptomatic in most individuals and disease typically arises when perturbation of host homeostasis and/or endogenous microbiota occurs. It was recently shown that administration of β-lactam antibiotics causes the release of bacterial peptidoglycan subunits—including tracheal cytotoxin among others—that induces the invasive hyphal fungal program in the gut [24]. Such antibiotic-induced pertrubations, immune system abnormalities (see below), changes in the microbiome, and/or changes in mucocutaneous barrier integrity [25,26] enable *C. albicans* to become an opportunistic pathogen in the context of expression of an array of virulence determinants (Table 1).

**Table 1.** Key *C. albicans-*associated virulence factors

Immune resistance and adaptation		
Adhesion/invasion	Associated genes	Function
	*ALS1*	Adhesin
	*ALS3*	Adhesin
	*SSA1*	AMP binding protein
	*HWP*	Hyphal-associated GPI-linked protein
	*AlA1*	Fibronectin-binding adhesin
	*MNT1* and *MNT2*	Involved in O-glycosylation
	*INT1*	Integrin-like protein involved in adhesion
Phenotypic switch	Associated genes	Function
	*CPH1*	Transcription factor
	*EFG1*	Transcription factor
	*INT1*	Filamentation inducer
	*TUP1*	Filamentation inducer
	*CZF1*	Hyphal growth
	*TEC1*	Filamentation inducer
Proteases	Associated genes	Function
	Secreted aspartyl proteinases *(SAP1-10)*	Secreted proteases
	Phospholipases *(PLB1-4)*	Phospholipases that cause disruption of host membranes
	*Lipases (LIP1–10)*	Lipases
Nutrient acquisition	Associated genes	Function
	*RBT5*	Heme-binding protein
	*CSA1-2*	Heme-binding proteins
	*PGA7*	Heme-binding protein
	*ZRT1*	Zinc transporter
	*PRA1*	Zinc acquisition
Environmental adaptation		
Biofilm formation	Associated genes	Function
	*BCR1*	Transcription factor
	*TEC1*	Transcription factor
	*EFG1*	Transcription factor
	*MKC1*	Maintains cellular integrity and cell wall biogenesis
pH sensing	Associated genes	Function
	*PHR1-2*	pH regulated genes that contribute to cell wall assembly and morphogenesis
	*RIM101*	pH response pathway
	*DFG16*	Plasma membrane receptor
	*RIM21*	Plasma membrane receptor
Thigmotropism	Associated genes	Function
	*CCH1*	Calcium channel
	*MID1*	Calcium channel
	*RSR1*	Hyphal orientation
Stress response	Associated genes	Function
	*GPP1-2*	Glycerol biosynthesis
	SOD	Superoxide dismutase
	*CTA1*	Catalase
	*YNB1*	Nitrosative stress response
	*HOG1*	Osmotic, oxidative and thermal stress response
	*HSP genes*	Heat shock and oxidative stress
Drug resistance		
	Associated genes	Function
	*CDR1* and *CDR2*	Transporter of the ATP binding cassette superfamily
	*TAC1*	Involved in the regulation of CDR1
	*MRR1*	Multidrug resistance regulator involved in the control of MDR1
	*FKS*	Encodes the β-(1,3)-d-glucan synthase involved in echinocandin resistance
	*MSH2*	DNA mismatch repair ATPase
	*HSP90*	Heat shock chaperone
	*UPC2*	Transcription factor associated with azole resistance
	*ERG3*	Sterol desaturase associated with azole and AMB resistance
	*ERG11*	Cytochrome P450 protein involved in demethylation of lanosterol, which upon modification it confers resistance to azoles
	*ERG6*	Methyltransferase, which converts zymosterol to fecosterol and is important for azole and AMB resistance
	*FCY2*	Cytosine permease involved in 5-FC resistance
	*FCY1*	Cytosine deaminase involved in 5-FC resistance
Immune evasion		
	Associated genes	Function
	*PRA1*	Complement binding protein
	*CRZ1*	Transcription factor. Crz1-dependent pathway activation induces lactate-induced β-glucan masking
	*ACE*	Transcription factor. Ace2 activation is involved in the network of lactate-induced β-glucan masking
	*XOG1*	Exoglucanase involved in immune evasion
	*ECE1*	Expressed in association with hyphae. Protein precursor of candidalysin

## Fungal virulence determinants in *C. albicans*

Plasticity in switching between different morphogenic states

*C. albicans* displays a range of cellular growth states that help it establish presence and persistence in different mammalian tissues [27]. These cell phenotypic variations are associated with different yeast-like or filament-like morphologies and colony features as well as distinct genetic profiles [27]. Especially important in the pathogenic life cycle of *C. albicans* is its ability to change morphology to and from the yeast and hyphal forms to breach mucosal barriers and establish invasive disease. Typically thought of as the commensal form, the yeast, allows colonization of superficial commensal niches [28,29]. By contrast, the hyphal form is typically thought of as the invasive form of the fungus allowing *C. albicans* to penetrate host barriers and to gain access into deep-seated tissues [30]. Several environmental stimuli affect the morphological state of *C. albicans* including host temperature, pH, nutrient availability, or quorum sensing mechanisms [31–34]. Of interest, both *C. albicans* yeast and hyphal morphotypes can be found in different anatomical areas across the mouse gut [35].

The importance of the morphogenic transition from yeast to hyphae is shown by the fact that nonfilamentous *C. albicans* strains are avirulent [36]. However, the evolutionary success and ability to cause life-threatening human disease of other non-*albicans Candida* species that are unable to filament such as C. *glabrata* and *C. auris* indicates that filamentation is not a prerequisite for pathogenesis in *Candida* species. In fact, hyphae-locked *C. albicans* strains are also hypovirulent *in vivo*, indicating that the transition between the yeast and filamentous forms is most critical for effective virulence, rather than each of the morphogenic states itself [37]. In addition, both *C. tropicalis* and *C. parapsilosis* strains engineered to exhibit hyper-filamentation phenotypes due to constitutively expression of the transcriptional regulator *UME6* showed a dramatic reduction in organ fungal burden during *in vivo* infection [38]. Notably, a recent report showed that metabolic adaptability and improved fitness led to enhanced fungal proliferation, which increased the virulence of filament-deficient strains in a mouse model of systemic candidiasis, when low fungal inocula were used. Interestingly, filament-deficient strains remained attenuated during intraperitoneal mouse infection, highlighting the tissue-specific cues that may affect the role of *C. albicans* morphogenic state and its virulence [39]. The ability of *C. albicans* to produce filaments *in vivo* during systemic candidiasis in mice correlates with the ability of the tissue to control infection. Thus, *C. albicans* produces filaments in the mouse kidney during systemic candidiasis whereas filamentation is not observed in the liver or spleen; this tissue-specific propensity to filament correlates with the ability of spleen and liver to control the infection as opposed to the kidney that is unable to control fungal proliferation and inexorably loses function [40]. Taken together, these observations underscore the critical contribution of the yeast-to-hyphae switch in *C. albicans* virulence and reinforce the importance of cell-intrinsic and tissue-specific environmental factors in the determination of the *C. albicans* morphotype under various niches and conditions.

Adhesion, invasion, and host cell damage^1^1.In all subheadings, the PDF proof does not have a line to separate the previous from the next subsection. please add a line of separation to make it easy for the reader to discern when a segment changes-throughout the paper

The importance of adhesion and invasion factors for the success of *C. albicans* as a pathogen is highlighted by the different types of surfaces, ranging from host mucosal tissues to medical devices and instruments, which *C. albicans* can colonize. In tissue, *C. albicans* yeast cells adhere to the epithelium and/or endothelium and trigger hyphal elongation with subsequent active penetration of host cells. The process is mediated by adhesin and invasin members of the Als and Hwp1 families (reviewed in detail elsewhere) [41–43].

During mucosal infection, the interaction between epithelial cells and fungal ligands leads to induced endocytosis and active penetration by *C. albicans*. During systemic infection, active penetration can give access to blood vessels via which fungal cells reach distant body sites. Thereafter, endothelial penetration initiates colonization and disseminated disease [44]. Induced endocytosis is mediated by the adhesins and invasins Als3 and Ssa1, which bind host cell N-cadherin on endothelial cells and E-cadherin on oral epithelial cells [45]. In oral epithelial cells, induced endocytosis activates platelet-derived growth factor BB (PDGF BB) and neural precursor cell expressed developmentally down-regulated protein 9 (NEDD9) signaling [46]. In addition, Als3 and Ssa1 interact with epidermal growth factor receptor (Egfr) and Her2 (also known as Erbb2) and both receptors function cooperatively to induce *C. albicans* hyphae endocytosis [47]. Adhesion of *C. albicans* to epithelial cells is followed by hyphal formation, which can then actively penetrate the plasma membrane of epithelial cells [48,49]. The formation of hyphae is accompanied by the expression of hypha-associated proteins with known damage and immune activation capabilities [50]. Moreover, secretion of hydrolases by *C. albicans* hyphae facilitates active penetration into epithelia contributing to extracellular nutrient acquisition [51]. *C. albicans* expresses three different classes of secreted hydrolases: proteinases, phospholipases, and lipases. Secreted aspartic proteinases (Saps) comprise ten members of which some are secreted (Sap1–8) and some remain bound to the cell surface (Sap9–10) [52]. Saps have been shown to play pleiotropic roles *in vitro* and *in vivo* including inducing damage to epithelial cells thus promoting fungal virulence, recruitment of neutrophils, and induction of pro-inflammatory responses such as IL-1β and TNF-α [53–56]. Phospholipases are extracellularly secreted and act via disruption of host cell membranes [57]. Lipases consist of 10 members (Lip1–10) and promote virulence in a mouse model of systemic candidiasis [58,59].

Candidalysin is a newly discovered hyphae-derived peptide toxin that has recently been shown to be a major virulence determinant of *C. albicans*. Hyphal filaments express *ECE1*, which encodes the Ece1p protein. Ece1p is processed by Kex2p and Kex1p to generate mature candidalysin that is then secreted. At high concentrations, candidalysin interacts with cell membranes to form pore-like structures resulting in membrane damage [60,61]. The resultant calcium influx and oxidative stress induced in host cells result in rapid necrotic—rather than apoptotic—cell death [62]. Additionally, Als3-mediated endocytosis of hyphal filaments leads to the formation of an endocytic vacuole with a high concentration of candidalysin potentiating the damage on oral epithelial cells [63,64]. A possible mechanism of immune protection against prolonged candidalysin damage involves neutralization of the toxin by albumin, which acts as an anti-toxin through hydrophobic interactions [65]. Notably, besides being essential for epithelial cell damage, candidalysin is also important for activation of mucosal and tissue-specific systemic immune responses (see below).

Metabolic adaptation and nutrient acquisition

In most settings, glucose is the preferred carbon source for *C. albicans* [66]. However, in most anatomical sites of colonization, fungal growth occurs under glucose-limiting conditions. Under those circumstances, C. *albicans* adapts by upregulating alternative carbon utilization pathways, using carboxylic acids such as lactate, amino acids, and N-acetylglucosamine (GlcNAc) [67]. C. *albicans* mutant strains in these pathways have attenuated virulence [68,69].

Tissues can be a limiting source of nutrients to the fungus. In addition, the host can withhold trace nutrients from microbes via nutritional immunity [70,71]. Thus, *C. albicans* has evolved strategies for acquisition of scarcely available micronutrients. Under zinc limitation, *C. albicans* produces a zinc-binding protein, encoded by *PRA1*, which scavenges zinc from host tissues [72]. Interestingly, competition for scarce micronutrients also influences the host immune response. Secretion of Pra1 disrupts host defense by blocking the complement component C3 and subsequent fungal clearance [73]. Zinc limitation in *C. albicans* induces a hyper-adherent phenotype termed Goliath cells [74,75]. In addition, dynamic changes in copper assimilation during systemic infection contribute to pathogenesis. Up-regulation of the copper efflux pump, Crp1, and the copper importer, Ctr1, is observed in a sequential, temporally regulated, manner during renal candidiasis and both factors are essential for fungal virulence [76,77]. During invasive infection, iron homeostasis is also perturbed triggering changes in the renal iron landscape. Thus, in the kidney medulla iron accumulates, whereas in the renal cortex leukocyte infiltrates form iron exclusion zones around fungal lesions [71]. Under low iron conditions, *C. albicans FTR1* acts as a high-affinity iron permease to promote iron uptake from ferritin and transferrin and is essential for virulence during systemic candidiasis [78]. Moreover, the adhesin and invasin Als3 was shown to promote iron uptake from ferritin in the context of *C. albicans* interactions with oral epithelial cells [79].

Stress resistance

The multitude of stressors imposed on *C. albicans* by host immune cells and the various microenvironment cues require the induction of differential stress resistance responses by *C. albicans*. For example, to escape oxidative killing by immune cells, *C. albicans* possesses six superoxide dismutases (Sods), which are all involved in the detoxification of reactive oxygen species (ROS) by converting O_2_^−^ into molecular oxygen and hydrogen peroxide [80]. Accordingly, Sod-deficient *C. albicans* strains have reduced pathogenicity [81]. Additional fungal antioxidant proteins and DNA damage repair genes help attenuate oxidative stress [82,83].

Other stressors include pH changes, thermal and osmolarity shifts, nitrosative stress, and antifungal drug treatment [84–86]. Many of the fungal stress responses are modulated by heat shock proteins (Hsps), particularly Hsp90. Hsp90 is a ubiquitous and conserved ATP-dependent molecular chaperone that acts to stabilize diverse signal transducers. *C. albicans* Hsp90 enables fungal virulence and drug resistance. This effect is mediated via modulation of the mitogen activated kinase Mck1, the stress activated protein kinase Hog1/Sty1, and/or the protein phosphatase calcineurin [87]. Additional effects of Hsp90 disruption include reduction of tolerance to stress responses and induction of morphological transition from yeast to hyphal growth [87].

Masking of cell wall components for immune evasion

**Figure 2.**
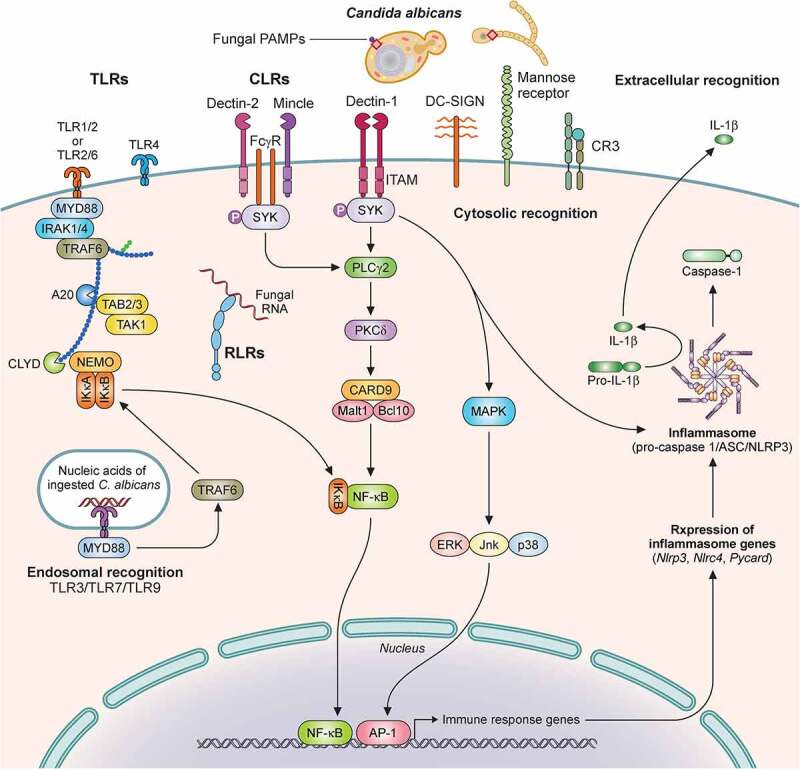
Recognition of *C. albicans* by immune cells is mediated by distinct pattern recognition receptor signaling pathways. Extracellular recognition of fungal ligands occurs via C-lectin receptors (CLRs) or by some of the Toll-like receptors (TLRs) such as TLR1, TLR2, TLR6, or TLR4. Recognition leads to the activation of intracellular signaling pathways dependent on several adaptor molecules inducing NF-κB activation and cytokine secretion. In addition, CLRs can activate AP-1 via MAPK also leading to cytokine secretion. Some TLRs (TLR3, TLR7, TLR9) recognize nucleic acids derived from *C. albicans* within the endosome. Within the cytosol, recognition is mediated by NOD-like Receptors (NLRs) with resultant inflammasome activation and IL‐1β processing. Recognition by TLRs and CLRs and secretion of pro-IL‐1β and pro-IL-18 triggers inflammasome assembly, activation of pro-caspase 1 to generate caspase-1, and cleavage of these two cytokines into their mature IL‐1β and IL-18 forms. Additional cytosolic recognition may occur via RIG-I-like receptors. Illustration created with BioRender.com. TLR, Toll like receptor; MyD88, Myeloid differentiation factor 88; IRAK1, Interleukin 1 receptor associated kinase 1; TAK1, transforming growth factor-β-activated kinase 1; TRAF, Tumor necrosis factor receptor-associated factor; TAB, TGF-beta-activated kinase; NEMO, nuclear factor-κB essential modulator; IκB kinase; CLYD, cylindromatosis tumor suppressor; RLR, RIG-I-like receptor; CLR, C-lectin receptor; Mincle, macrophage inducible Ca2+-dependent lectin receptor; FcγR, Fc receptors: SYK, Spleen tyrosine kinase; ITAM, immunoreceptor tyrosine based activation motif; PLCγ2 Phospholipase C gamma 2; PKCδ, Protein kinase C delta; CARD9, caspase recruitment domain-containing protein 9; Malt1, mucosa-associated lymphoid tissue lymphoma translocation 1; NF-κB, nuclear factor kappa-light-chain-enhancer of activated B cells; DC-SIGN, Dendritic cell-specific intercellular adhesion molecule-3-Grabbing non-integrin; CR3, Complement receptor 3; MAPK, mitogen-activated protein kinase; ERK, Extracellular signal-regulated kinase; JNK, c-Jun N-terminal kinase; AP-1, activator protein-1; ASC, Apoptosis-associated speck-like protein containing a CARD; NLRP3, NLR family pyrin domain containing 3; NLRC4, NLR family CARD domain containing 4.

The first step in the development of an immune response to *C. albicans* is the recognition of fungal pathogen-associated molecular patterns (PAMPs) by host epithelial and immune cell pattern recognition receptors (PRRs) (Figure 2). The *C. albicans* cell wall is composed of chitin, β-glucan, and mannoproteins [88], which are recognized by host cell PRRs and are important for induction of protective antifungal immune responses.

To avoid activating immune pathways that trigger these effector responses, *C. albicans* cells can minimize their PAMP exposure by masking cell wall components in response to metabolic cues. These protective responses circumvent the immune system by exploiting host signals to promote immune evasion [89]. Some of these signals include changes in oxygen availability, carbon source, or hormone levels [90–96]. The architecture of the fungal cell wall can also be altered by environmental stress during infection favoring immune recognition, as is the case for pH or iron changes [97,98]. For example, high iron decreases the levels of mannans and chitin and increases the levels of β-(1,3)-glucan by preventing activation of the fungal mitogen-activated protein kinase (MAPK) Choline/ethanolamine kinase 1 (Cek1), an effect mediated by lactate-induced Crz1 [98]. These changes reduce the susceptibility of *C. albicans* to cell wall-perturbing antifungal agents but also reduce survival upon phagocytosis by macrophages. Remarkably, treatment with sub-therapeutic doses of the antifungal drug caspofungin also causes exposure of *C. albicans* β-glucan both *in vitro* and *in vivo* and elicits pro-inflammatory cytokine secretion by primary macrophages [99]. This unmasking is modulated by changes in the regulatory gene network responsible for the cell wall architecture [100].

To avoid clearance within phagosomes, *C. albicans* activate programs to survive the nutrient-poor, acid environment. To adapt to this nutrient-poor niche, fungal cells induce metabolic starvation pathways, including gluconeogenesis, fatty acid degradation, and also downregulate translation [101]. To induce filamentation, yeast cells produce ammonia, which promotes neutralization of the acidic phagosomal pH and yeast-to-hyphae transition [102,103].

## Initiation of host responses against *C. albicans –* the role of PRR systems

Central to the initiation of antifungal immune responses are the members of the C-type lectin (CLR) superfamily. Since the discovery of the role of DECTIN-1 in the recognition of fungal β-glucan [104,105], the characterization of other members of the CLR family, including—but not limited to—DECTIN-2 [106], DECTIN-3 [107], and MINCLE [108] has uncovered the importance of CLR-mediated signaling in innate immune sensing and control of pathogenic fungi, including *C. albicans* [109,110].

Fungal sensing via CLRs is the first step in the activation of the CLR–spleen tyrosine kinase (SYK)–caspase recruitment domain-containing protein 9 (CARD9) signaling pathway [109,110]. Following fungal ligand recognition by the corresponding CLR, its hemITAM or ITAM— depending on the CLR—is phosphorylated and SYK is activated, which leads to assembly of the CARD9-BCL10-MALT1 complex. This engagement activates the canonical nuclear factor kappa-light-chain-enhancer of activated B cells (NF-κB) subunits c-Rel and p65 and induces innate and adaptive immune responses and cytokine secretion. Noncanonical NF-κB activation can also be induced in a SYK/NF-κB-inducing kinase (NIK)-dependent manner [110]. SYK-deficient mice are susceptible to systemic candidiasis (and other fungal infections) and SYK-deficient neutrophils are unable to control several *Candida* species associated with defects in ROS production, cytokine production, neutrophil extracellular trap (NET) formation, phagocytosis, and neutrophil swarming [111,112]. SYK integrates signals from multiple CLR-dependent and CLR-independent signaling pathways, thus, SYK activation requires a delicate balance whereby a suboptimal response can cause immunodeficiency, whereas an excessive response can lead to hyper-inflammatory disease and hematological malignancy [113,114].

The critical contribution of the CLR–SYK–CARD9 signaling pathway in human antifungal host defense is clearly portrayed by the observation that CARD9 deficiency is the only— among the >400 known to date—primary immunodeficiency disorder (PID) that promotes fungal-specific infection susceptibility without predisposition to bacterial, viral, or parasitic infections or noninfectious complications. In fact, CARD9 deficiency is the only known inherited condition that underlies susceptibility to both mucosal and systemic candidiasis, with the latter exhibiting a unique predilection for the CNS [115]. Aspergillosis—primarily extrapulmonary—, phaeohyphomycosis, and deep-seated dermatophytosis also occur in CARD9-deficient patients [116–119]. Critical CARD9-dependent fungal surveillance immune functions include Th17 cell differentiation, neutrophil recruitment to the CNS, pro-inflammatory cytokine and chemokine production, and phagocyte fungal killing [120–123]. The advent of the SYK inhibitor fostamatinib in clinical practice for the treatment of various inflammatory and neoplastic conditions may result in opportunistic mucosal and/or systemic fungal infections, as indicated by the early description of mucosal candidiasis and skin fungal disease in fostamatinib-treated individuals [114,124,125].

CARD9 relays signals from several upstream CLRs in a fungus- and tissue-specific manner, without a single CLR deficiency phenocopying CARD9 deficiency. For example, a few DECTIN-1–deficient patients who carry the p.Y238* *CLEC7A* mutation in homozygosity [126], which abolishes DECTIN-1–dependent signaling, were reported to develop recurrent VVC (RVVC) and onychomycosis. In addition, a single immunocompromised DECTIN-2–deficient patient carrying a homozygous deletion resulting in a frameshift and early stop codon in DECTIN-2 was recently reported to develop fatal invasive pulmonary aspergillosis [127]. As mentioned earlier, the *C. albicans* cell wall structure is dynamically altered during infection and therefore immune recognition often requires the concerted action of several PRRs to mount effective immune responses; among others, such interactions have been characterized between different CLRs, between CLRs and TLRs, and between TLRs and the complement C5a anaphylatoxin [4,128–130].

Toll-like receptors (TLRs) are expressed on both hematopoietic and non-hematopoietic cells and also participate in *C. albicans* sensing. Upon fungal PAMP recognition, MYD88 is recruited to the TLR and a signaling cascade is initiated that culminates in pro-inflammatory cytokine and chemokine production in a NF-κB dependent manner. MYD88 is required for activation of Langerhans cells and induction of the Th17 response during skin *Candida* infection (see below) [131]. Moreover, *Myd88*^−/−^ mice are susceptible to systemic fungal infections—including systemic candidiasis—however MYD88-deficient patients do not develop candidiasis or other fungal disease, likely due to compensatory effects of other PRRs, primarily of CLRs [132,133].

TLR2 recognizes *C. albicans* phospholipomannans and TLR2 deficiency impairs neutrophil chemotaxis, phagocytic activity, and cytokine and chemokine production resulting in reduced survival during systemic candidiasis in mice [134,135]. However, other studies have shown a dispensable role for TLR2 during systemic candidiasis, which may be explained by the differential dependence of different *C. albicans* strains on TLR2 recognition [136]. Indeed, *C. albicans* strain-specific differential dependence on PRR recognition has also been documented for DECTIN-1 and TLR4, which recognizes *C. albicans* O-linked mannans [132,137–141]; in the setting of candidiasis *in vivo*, these differences underlie a wide variety of outcomes ranging from conferring survival benefit to promoting lethal immunopathology. In mice, TLR1 deficiency increased whereas TLR6 deficiency ameliorated intestinal inflammation and *C. albicans* burden in a colitis model [142]. In humans, polymorphisms in *TLR1, TLR4*, and *TLR6* have been suggested to confer greater susceptibility to systemic candidiasis in acutely ill patients in the intensive care unit (ICU) in some studies [143–145]. *C. albicans* DNA sensing by TLR9 promotes IL-12p40 production [146], and TLR9—together with the mannose receptor and NOD2—has also been shown to recognize *C. albicans* chitin leading to the production of the anti-inflammatory cytokine IL-10 [147]. Moreover, the endosomal TLR7 and TLR3 have been implicated in *C. albicans* RNA sensing leading to the production of type I interferons and pro-inflammatory chemokines [148–150].

NOD-like receptors (NLRs) are cytoplasmic PRRs that enable the formation of inflammasomes, which are multiprotein complexes that process pro-IL-1β/pro-IL-18 into their mature forms. The NLRP3 inflammasome complex is formed by NLRP3, the adaptor protein ASC, and the effector caspase-1, although non-canonical caspase-8-dependent pro-IL-1β processing is also operational in *C. albicans* [151]. The morphological switch of *C. albicans* contributes to NLRP3 activation in a TLR2/DECTIN-1/SYK-dependent manner [152–154]. Mice deficient in NLRP3, ASC, or caspase-1 have increased fungal proliferation and decreased survival during systemic candidiasis [152,154]. Moreover, the NLR family member NLRP10 was critical for survival during systemic candidiasis in mice via promoting Th1 and Th17 responses, while being dispensable for pro-inflammatory cytokine production [155]. In addition, the NLRC4 inflammasome is important for the control of mucosal candidiasis *in vivo*. Specifically, NLRC4 is upregulated in oral mucosal tissues following *C. albicans* infection, particularly in the stromal compartment, it mediates—together with NLRP3—the induction of IL-1β, and NLRC4-deficient mice had reduced secretion of CRAMP, IL-17A, and IL-1β, and impaired ability to control mucosal fungal proliferation [156].

Other receptors that have been implicated in the initiation of immune responses against *C. albicans* include: a) the RIG-I-like receptor family receptor MDA5 (IFIHI), which plays a major role in viral RNA sensing [157], is induced in response to *C. albicans* hyphae, and may be dysfunctional during mucosal and systemic candidiasis in susceptible patients [158], and b) EphA2, a receptor tyrosine kinase present in epithelial cells, which mediates fungal β-glucan recognition and induces pro-inflammatory responses during OPC [159]. In addition, expression of EphA2 on neutrophils is important for immunity during OPC via MEK-ERK signaling and subsequent priming of nicotinamide adenine dinucleotide phosphate (NADPH)-subunit p47phox and ROS production, which results in fungal killing [160].

Following initial sensing by the innate immune system, host immune responses are deployed during mucocutaneous and systemic candidiasis; these responses are tissue-specific, compartmentalized, and distinct in the various forms of the infection. The cellular and molecular basis of these responses is briefly outlined in the next section.

## Mucocutaneous candidiasis

OPC and EPC

Candidiasis of the mouth—primarily affecting the tongue, buccal mucosa, and gingivae—, throat, or esophagus is predominantly caused by *C. albicans* and is uncommon in healthy individuals. HIV/AIDS is a major risk factor for OPC and EPC, typically in patients with diminished CD4 T cell counts (<200 cells/mm^3^), whereas certain topical or systemic immunosuppressive agents also predispose to the infection such as corticosteroids [161], TNF-α inhibitors [162], and IL-17-targeted biologics (see below) [163]. Other local factors that contribute to OPC susceptibility include denture wearing and salivary hypofunction; salivary flow acts as a mechanical clearance mechanism by preventing adherence of *C. albicans* to oral epithelial cells and saliva contains potent AMPs with *C. albicans*-inhibitory properties (see below) [164].

The critical pathway mediating oral mucosal immune protection is IL-17 signaling [165]. Following the initial seminal reports of mice deficient in IL-17RA, IL-17RC, or the IL-17 receptor adaptor ACT1 being highly susceptible to OPC, subsequent studies in patients confirmed the critical contribution of this signaling axis in mucosal anti-*Candida* host defense [166–168]. Thus, patients with autosomal recessive complete deficiencies in IL-17RA, IL17RC, or ACT1/TRAF3IP2 develop fully penetrant, severe, treatment-refractory mucosal infections by *Candida* species, termed chronic mucocutaneous candidiasis (CMC) [169–172]. A single kindred carrying a heterozygous dominant-negative mutation in *IL17F* that impaired cellular responses to both IL17F and IL17AF was also reported to result in CMC, yet with incomplete penetrance [172]. Other inborn errors of immunity manifesting with CMC also map to defects in IL-17 signaling featuring varying degrees of decreased frequencies of circulating Th17 cells and/or impaired IL-17 cellular responses, further illustrating its importance for mucosal antifungal protection (Table 2) [173–178]. More recently, the use of IL-17 pathway-blocking monoclonal antibodies (mAbs) in patients with psoriasis and inflammatory bowel disease (IBD) has been associated with the development in some patients with mild, treatment-responsive OPC, but not CMC. Notably, the mean frequency of OPC in these patients is low (~1-10%), with a greater risk observed in patients receiving mAbs that target IL-17RA or combined IL-17A, IL-17F, and IL-17AF, followed by mAbs that target IL-17A, followed by mAbs that target IL-12p40 or IL-23p19 [163]. The resistance of these patients to CMC likely reflects the incomplete blockade of mucocutaneous IL-17 signaling by the administered mAbs [179,180]. Collectively, these data indicate that a complete absence of IL-17R responses promotes susceptibility to CMC in humans, whereas an mAb-induced blockade regimen that spares a fraction of mucosal IL-17R responses does not.^2^2.In Table 2, in the PDF Proof (not in here), there are words that belong in the above sentence that are shown in the sentence below. Eg in IL-12p40 deficiency NTM and infections are separately by a line and they shouldn't. Same for LAD-1 where gram-negative and bacteria, periodontitis are separately by a line. Also there is an indent in the genes listed for SCID in the PDF and in the genes listed for CGD. they should all be listed without an indent.

**Table 2.** Inborn errors of immunity underlying inherited susceptibility to *C. albicans* infections

Primary immunodeficiency disorder	Associated gene	Mode of inheritance	Clinical presentation of infection
Mucosal candidiasis			
APECED	*AIRE*	AR or AD	CMC
DOCK8 deficiency	*DOCK8*	AR	CMC, viral infection (molluscum, HSV)
ZNF341 deficiency	*ZNF341*	AR	CMC, bacterial infections (sinopulmonary and skin bacterial infections)
JNK1 haploinsufficiency	*MAPK8*	AD	CMC, superficial skin bacterial infections
IRF8 deficiency	*IRF8*	AR	CMC, disseminated NTM infection
RORγt deficiency	*RORC*	AR	CMC, disseminated NTM infection
IL-12p40 deficiency	*IL12B*	AR	CMC, intracellular bacterial and NTM infections
IL12Rβ1 deficiency	*IL12RB1*	AR	CMC, intracellular bacterial and NTM infections
IL-17RA deficiency	*IL17RA*	AR	CMC, bacterial infections (superficial staphylococcal skin infections, bacterial pneumonias)
IL-17RC deficiency	*IL17RC*	AR	CMC
IL-17F deficiency	*IL17F*	AR	CMC
ACT1 deficiency	*TRAF3IP2*	AR	CMC, bacterial infections (superficial staphylococcal skin infections, bacterial pneumonias)
Job’s syndrome	*STAT3*	AD	CMC, onychomycosis, pulmonary mold infections, skin and pulmonary bacterial infections
STAT1 gain-of-function	*STAT1*	AD	CMC, bacterial and NTM infections, viral infections, endemic fungal infections
SCID	*IL7RA* *IL2RG* *RAG1-2* *JAK3*	AR	CMC, bacterial infections, disseminated viral infections, PJP
EDA-ID	*KBKG* *IKBA*	AR	CMC, NTM infections
Systemic candidiasis			
CGD	*CYBA* *CYBB* *NFC1* *NFC2*	AR or X-linked	invasive mold infections, systemic candidiasis (rare), invasive bacterial infections (*Staphylococcus, Nocardia, Serratia*)
LAD-1	*ITGB2*	AR	Systemic candidiasis, pyogenic bacterial infections (staphylococcal skin infections and gram negative bacteria, periodontitis)
Complete MPO deficiency	*MPO*	AR	Systemic candidiasis
Mucosal and systemic candidiasis			
CARD9 deficiency	*CARD9*	AR	CMC, *Candida* meningitis, colitis, endophthalmitis, and osteomyelitis, aspergillosis (including extrapulmonary), phaeohyphomycosis, protothecosis

AD, autosomal dominant; AR, autosomal recessive; APECED, autoimmune polyendocrinopathy-candidiasis-ectodermal dystrophy; HSV, herpes simplex virus; NTM, nontuberculous mycobacteria; CMC, chronic mucocutaneous candidiasis; PJP, *Pneumocystis jirovecii* pneumonia; SCID, severe-combined immunodeficiency disorder; EDA-ID, anhidrotic ectodermal dysplasia with immunodeficiency: CGD chronic granulomatous disease; LAD-1, leukocyte adhesion deficiency type-1; MPO, myeloperoxidase; AIRE, autoimmune regulator; CARD9, caspase recruitment domain-containing protein 9; STAT, signal transducer and activator of transcription; DOCK8, dedicator of cytokinesis 8.

At the cellular level, CD4^+^ T cells, CD8^+^ T cells, γδ T cells, and type 3 innate lymphoid cells (ILC3) are the major sources of IL-17 during oral candidiasis [181,182]. Initial innate Th17 responses are regulated by Langerin-expressing dendritic cells (DCs) and are deployed by rapidly proliferating tissue-resident natural Th17 (nTh17) cells post-*C. albicans* challenge [182–184]. The development of *C. albicans*-specific Th17 cells following OPC involves antigen presentation and T cell priming by tissue-resident DCs in an Flt3L-dependent manner aided by monocyte-derived DCs in a CCR2-dependent manner [185]. *C. albicans*-specific IL-17-producing tissue-resident memory T cells (T_RM_) are efficient in maintaining prolonged colonization in a mouse model of *C. albicans* commensalism [186,187].

Of note, data from mice and humans suggest that the lack of IL-17 production by a certain lymphoid cell subset, may be compensated by other IL-17-producing lymphoid cells. For example, *Tcrb*^−/−^ and *Tcrgd*^−/−^ mice eventually control OPC whereas *Rag1*^−/−^ mice that lack both αβ and γδ T cells are highly susceptible to the infection [188]. In agreement, patients with idiopathic CD4 lymphocytopenia who have diminished CD4^+^ T cells and patients with loss-of-expression mutations in the *CD4* gene who also lack Th17 cell-derived IL-17 production do not manifest CMC [189–191]. Collectively, these data indicate that the susceptibility of HIV/AIDS patients to OPC may reflect defects in IL-17 production by both Th17 and non-Th17 cellular sources at the oral mucosa, as suggested by studies in SIV-infected non-human primates [192–194].

Mechanistically, IL-17 mediates a robust mucosal immune response to protect against *C. albicans* by acting on IL-17R-expressing epithelial cells to induce the production of potent AMPs such as β-defensins and S100A8/A9 [165,168,195]. Accordingly, *Defb3*^−/−^ mice were susceptible to OPC [165]. Histatins are another important family of AMPs, which have been shown to prevent *C. albicans* colonization on epithelial cell surfaces, to protect the basal epithelial cell layer from apoptosis, and to alter *C. albicans* mitochondrial function resulting in fungal cell death [196–198]. IL-17 signaling also promotes the production of neutrophil-recruiting CXC chemokines (e.g. CXCL1, CXCL5) and has been shown to be indispensable for neutrophil recruitment in the *C. albicans*-infected oral mucosa in some—but not all—studies, potentially reflecting microbiome variations in the mouse colonies used in these different settings [195,199]. Mice lacking the CXCL1/CXCL5-targeted chemokine receptor CXCR2 were highly susceptible to OPC due to impaired neutrophil recruitment to the *C. albicans*-infected oral mucosa [200]. Besides IL-17R signaling, IL-1R signaling promotes mobilization of granulocytes from the bone marrow and neutrophil recruitment into the oral mucosa during OPC via endothelial cell production of granulocyte colony-stimulating factor (G-CSF) in response to keratinocyte-derived IL-1α [201].

IL-22 is another cytokine produced by type 17 innate and adaptive lymphoid cell subsets during OPC and plays an important role in antifungal resistance as shown by experiments in *Il22r*^−/−^ mice, in wild-type mice administered IL-22-targeted mAbs, and in mice deficient in both IL-17R and IL-22R, which exhibit further increase in fungal proliferation compared to mice deficient in either IL-17R or IL-22R [199,202]. Mechanistically, IL-22 acts on its receptor on oral basal epithelial cells to provide survival and regeneration signals to the IL-17R-expressing oral suprabasal epithelial cell layer enabling its responsiveness to IL-17A [199]. In humans, no inborn errors of IL-22 immunity have thus far been reported to cause CMC. Patients with loss-of-function mutations in the IL-22 receptor subunit IL-10RB, who lack IL-22 (and IL-10, IL-26, IL-28, and IFNL1) responses, do not develop CMC but manifest with very early onset IBD [203,204]. These data collectively indicate that IL-22 deficiency appears to be tolerated in humans and that impaired IL-22 responses may act synergically with defective IL-17 responses to cooperatively impair mucosal anti-*C. albicans* host defense.

On the fungal front, as described above, *C. albicans* adherence to oral epithelial cells is achieved via the Als and Hwp families of adhesins/invasins [41–43,205]. Fungal recognition activates NF-κB and a biphasic MAPK innate response in oral epithelial cells. Triggered by *C. albicans* cell wall recognition and independent of fungal morphology, the first step in the signaling cascade involves NF-κB and MAPK/c-Jun activation. The second MAPK phase occurs in response to a greater *C. albicans* burden and filament formation, with c-Fos and MKP1 activation leading to induction of pro-inflammatory responses [206]. This complex response helps oral epithelial cells to discriminate between colonizing and invading *C. albicans*. During OPC, candidalysin acts as a driver of protective IL-17 responses as well as of IL-36 induction via synergistic interactions between IL-1α and EGFR signaling in oral epithelial cells [183,207]. In addition, in a PAMP-independent manner, candidalysin induces EGFR phosphorylation leading to secretion of neutrophil-targeted chemokines [208]. In candidalysin-exposed epithelial cells, blockade of IL-1α/IL-1R resulted in decreased IκBα phosphorylation, reduced induction of IκBζ, and impaired production of granulocyte-macrophage colony-stimulating factor (GM-CSF) and neutrophil-recruiting IL-8/CXCL8. Combined blockade of EGFR and IL-1R further suppressed pro-inflammatory cytokine production in candidalysin-exposed cells [209].

Although type 17 immunity is undoubtedly critical for protective mucosal anti-*Candida* host defense in mice and humans, we recently reported that, in certain settings, additional, IL-17R/IL-22-independent mechanisms can also promote mucosal fungal infection susceptibility. We studied mice and humans with Autoimmune polyendocrinopathy–candidiasis–ectodermal dystrophy (APECED), also known as Autoimmune polyglandular syndrome type 1 (APS-1), a monogenic autoimmune disorder characterized by loss-of-function mutations in the autoimmune regulator (*AIRE*) gene [210]. APECED patients feature selective infection susceptibility to CMC with a frequency of ~80-90%, associated with serum autoantibodies against IL-17F (frequency, ~20-85% depending on the cohort), IL-17A (frequency, ~35%), and IL-22 (frequency, ~70-90% depending on the cohort) [211–214]. However, the association between these autoantibodies and CMC in APECED is incompletely penetrant, and several patients who carry these autoantibodies do not develop CMC, while several other patients who lack these autoantibodies manifest CMC [211–214]. These data indicate that additional factors must contribute to susceptibility to CMC in APECED patients.

Indeed, we probed oral mucosal immune responses in Aire-deficient mice, which exhibited selective infection susceptibility to CMC despite the fact that they rarely develop type 17 cytokine-targeted autoantibodies (frequency, <10%) and they mount intact IL-17R/IL-22 mucosal immune responses during OPC [215]. These data indicate that impaired type 17 immunity is not the primary driver of OPC susceptibility in Aire-deficient mice. Instead, the OPC susceptibility in Aire-deficient mice was driven by the overproduction of interferon-γ (IFN-γ) by oral mucosal CD4^+^ and CD8^+^ T cells, which were both necessary and sufficient to promote infection in this setting via disrupting the oral epithelial barrier. Accordingly, genetic or pharmacological inhibition of IFN-γ or JAK-STAT signaling rescued the epithelial barrier defects and reversed OPC susceptibility in Aire-deficient mice [215]. Moreover, we found corroborative evidence of excessive type 1 and intact type 17 immune responses in the oral mucosa of APECED patients [215]. Taken together, these data indicate that, in certain settings, aberrant type 1 mucosal responses rather than impaired type 17 mucosal responses may promote mucosal fungal susceptibility and that T cell-driven immunopathology rather than impaired host resistance may underlie mucosal candidiasis. Together, these findings point to a novel conceptual framework for classifying CMC molecular subtypes across a spectrum of defective type 17 mucosal defense and/or immunopathology-promoting type 1 mucosal inflammation [188].

More studies are needed to further evaluate the relative contribution of these pathways in the initiation, persistence, and/or recurrence of CMC in additional APECED children and adults and to determine whether aberrant type 1 mucosal responses may contribute to CMC in other conditions with excessive type 1 inflammation such as a) trisomy 21 in which circulating Th17 cells are intact and b) STAT1 gain-of-function in which several patients develop CMC despite normal circulating Th17 cells and intact production of IL-17 by circulating T cells following fungal-specific stimulation and in which JAK-STAT inhibitors ameliorate CMC [216–218]. Of note, in a different setting, autoreactive T cells were shown to promote chronic mucosal fungal infection in mice leading to excessive inflammation, epithelial injury, and esophageal squamous cell carcinoma development, which is a feature of certain CMC-manifesting immune dysregulatory PIDs such as APECED and STAT1 gain-of-function [216,219–221].

VVC

VVC will occur at least once in ~75% of the women worldwide during their reproductive years with 6-10% of them developing >4 recurrent infections per year, a condition termed RVVC [222,223]. VVC, caused predominantly by *C. albicans* but also by *C. glabrata, C. parapsilosis, C. krusei*, and *C. tropicalis*, is a debilitating condition with substantial prevalence, economic burden, and morbidity [222]. VVC is associated with aberrant vaginal inflammation triggered by the presence of *Candida* in the setting of local immune dysregulation, hormonal changes, vaginal microbiome alterations, and/or damaged mucosa [223,224]. Accordingly, uncontrolled diabetes mellitus, sexual activity, increased estrogen states such as during pregnancy and oral contraceptive or hormone replacement therapy, and antibiotic use are among the most common risk factors for VVC [222,225]. Although beta-lactams are more frequently implicated in the development of VVC relative to other classes of antibiotics, the mechanisms by which specific antibiotics perturb the local microbiome to enable vaginal fungal colonization and infection remain elusive [225,226].

Notably, although HIV/AIDS patients are highly susceptible to OPC and EPC, they are not at a greater risk for developing VVC and, congruently, lymphocyte depletion does not impair fungal control during experimental VVC in mice [227–229]. In addition, although IL-17R responses are induced after infection, the control of fungal proliferation is not reliant on the IL-17 signaling axis during VVC, and patients receiving IL-17 pathway-targeted mAbs have not been reported to be at a significant risk for VVC [163,230]. Taken together, these observations highlight the differential oral and vaginal mucosa-specific host immune requirements for anti-*Candida* protection.

Vaginal epithelial cells avert *C. albicans* adhesion to and invasion of the mucosa via shedding of the superficial epithelial layer into the vaginal lumen and coating of epithelial cells with mucin [231]. Upon fungal sensing, vaginal epithelial cells activate early mitochondrial signaling characterized by a protective type I interferon response that is shared between *C. albicans* and non-*albicans Candida* species (i.e. *C. glabrata, C. parapsilosis*, and *C. tropicalis*). This is followed by a subsequent damage response that is specific to *C. albicans* and is directed by the secretion of candidalysin [232]. Moreover, similar to oral epithelial cells, vaginal epithelial cells employ NF-κB activation and a biphasic MAPK response to discriminate between *C. albicans* yeast and hyphal morphotypes, albeit with delayed c-Jun activation and differential pro-inflammatory responses characterized by reduced secretion of IL-6, CCL20, and G-CSF [206,233]. In addition, RNA-seq analysis of patient samples indicated that target genes of the PDGF BB and ERBB2 pathways were up-regulated during VVC whereas target genes of the NEDD9 pathway were not, in contrast to their induction in oral epithelial cells [46].

Investigations in mouse models and humans with VVC including studies of intravaginal challenge with live *C. albicans* in healthy adult women [234] have established that neutrophils drive immunopathology and underlie VVC symptoms while they are ineffective in mediating fungal clearance in the vaginal microenvironment. In mice with VVC, neutrophil depletion ameliorated inflammation without increasing vaginal fungal load [235]. *C. albicans* virulence factors that trigger neutrophil recruitment in the vagina include candidalysin and Sap1 through Sap6. [56,236–239,236,240]. The transepithelial migration of neutrophils into the vagina is promoted via the CXCL1-CXCR2 chemokine axis and via estradiol receptor alpha-dependent epithelial expression of CD44 and CD47, both of which are modulated differentially by estrogen and progesterone [241,242]. Notably, recent studies have shed light on the mechanisms of neutrophil dysfunction within the vaginal milieu, termed “neutrophil anergy”; specifically, vaginal heparan sulfate was shown to act as a competitive ligand for Mac-1 on neutrophils, which inhibits their *Candida* binding and killing properties [243,244].

Integral to the immunopathogenesis of VVC is also inflammasome-primarily NLRP3-activation, and IL-1β production, associated with *C. albicans*-derived candidalysin and Saps [238,245–247]. *Nlrp3*^−/−^ mice have reduced neutrophil infiltration, alarmin production, and pro-inflammatory cytokine secretion in vaginal lavage fluid during VVC, and NLRP3 and caspase-1 are upregulated in women with VVC compared to asymptomatic women who were either *C. albicans*-colonized or non-colonized [238,248]. Correspondingly, the presence of the 12/9 genotype upon examination of a variable number tandem repeat polymorphism in the *NLRP3* gene was associated with increased susceptibility to RVVC and a greater production of IL-1β in the vagina [249]. Moreover, a polymorphism in the *SIGLEC15* gene, a lectin expressed by immune cells that binds sialic acid-containing structures, was associated with RVVC and correlated with increased *IL1B* and *NLRP3* expression after *Candida* stimulation [250]. Additional polymorphisms in PRR (*TLR2, CLEC7A*) and cytokine (*IL4*) genes have also been associated with the development of RVVC [251–253]. Importantly, IL-22 curtails NLRP3 inflammasome activation and neutrophil recruitment during VVC by inducing the NLRC4 inflammasome, which promotes the production of the IL-1 receptor antagonist (IL-1Ra) [254]. In a mouse model of VVC, recombinant IL-1Ra reduced NLRP3-driven inflammation and protected against *C. albicans* [254], as did boosting of the protective effects of IL-22 via engaging the aryl hydrocarbon receptor with indole-3-aldehyde, thus providing potential translational avenues for therapeutic intervention [255,256]. In addition, an *IL22* polymorphism that led to greater levels of IL-22 and decreased levels of pro-inflammatory cytokines in the vagina correlated with increased resistance to RVVC, as did an *IDO1* polymorphism, which was associated with greater vaginal *IDO1* expression, increased kynurenine levels, and higher IL-22 and decreased pro-inflammatory cytokine levels [253].

In the past decades, several groups have worked toward developing an anti-*Candida* vaccine, and VVC has been a major infection manifestation targeted for protection [257]. Promising preclinical data have been generated using vaccine candidates that target *C. albicans* β-glucan or Sap2 [258,259]. Yet, the most promising vaccine candidate to date, which has demonstrated efficacy in both preclinical models and in human clinical trials, is NDV-3A, which is based on the N-terminal portion of the *C. albicans* Als3 protein (rAls3p-N) with an alum adjuvant. In a mouse model of VVC, immunization with NDV-3A led to production of high-titer anti-rAls3p-N serum IgG and vaginal IgA antibodies, decreased neutrophil influx, and enhanced *C. albicans* killing by neutrophils, and protected against vaginal fungal proliferation in a manner dependent on both T and B lymphocytes [260]. In a Phase I clinical trial, administration of NDV-3A in healthy volunteers was safe and resulted in IgG and IgA antibody responses and in IFN-γ and IL-17A cellular responses [261]. In a Phase II, randomized, double-blinded, placebo-controlled clinical trial, administration of NDV-3A to women with RVVC was safe, highly immunogenic, and efficacious resulting in reduced frequency of symptomatic episodes of VVC, particularly in <40 year-old women [262]. Higher serum anti-rAls3p-N IgG titers—particularly of the IgG2 subclass—were observed in vaccinated women who did not experience VVC recurrence relative to those who recurred pointing to a potential surrogate immunological marker of vaccine efficacy [263].

Cutaneous *C. albicans* infections^3^3.as mentioned above, subsegments are separated well here but not on the PDF proof.

At the steady state, the human skin is colonized by diverse fungal species, predominantly *Malassezia*, whereas the abundance of *Candida* species increases dramatically in human skin with immune dysregulation and/or broad-spectrum antibiotic exposure [264–266]. The emerging multidrug-resistant *C. auris* is an efficient long-term colonizer of the mouse, porcine, and human skin—but not of the gastrointestinal tract in contrast to *C. albicans* [5,267,268]. *C. albicans*—but also *C. tropicalis, C. parapsilosis*, and other *Candida* species—can cause clinical mucocutaneous disease in the forms of onychomycosis, paronychia, diaper rash, balanitis—often in uncontrolled diabetes mellitus, or other cutaneous infections [269,270].

The outer layer of the epidermis—the stratum corneum—is a cornified envelope composed of dead keratinocytes, keratin, and lipids including ceramides with ultra-long-chain acyl moieties, which create a dense physical barrier against potential pathogens such as *C. albicans*. Mice deficient in ceramide synthase 3 have a defective cornified lipid envelope and disrupted cutaneous barrier function and are susceptible to *C. albicans* skin infection [271]. Underneath the stratum corneum, the granular, spinous, and basal layers of the skin epidermis contain live keratinocytes to which *C. albicans* adheres via interactions of fungal phosphoglycerate mutase (Gpm1) with epithelial cell vitronectin [272]. CLR- and TLR-expressing keratinocytes constitutively express IL-17R via which they respond to IL-17 to generate AMPs for achieving fungal clearance (see below). Moreover, melanocytes located in the basal layer of the epidermis synthesize melanin, which has antimicrobial properties, and recognize *C. albicans* via TLR4 to increase melanization and exert an inhibitory fungal effect [273,274].

Cutaneous nerve fibers in the epidermis and dermis, particularly those expressing the neuropeptide calcitonin gene-related peptide (CGRP) which is known to mediate pain signaling, have also been shown to participate in protective cutaneous responses against *C. albicans* through direct antifungal properties of CGRP, and by promoting keratinocyte proliferation and regulating IL-23 production by dermal DCs (see below) [275]. Sensory neurons are activated by *C. albicans* and their mechanical ablation or chemical denervation of TRPV1^+^ neurons impaired IL-23 and IL-17 responses and increased susceptibility to cutaneous *C. albicans* infection, which was rescued by the addition of CGRP [276]. In fact, activation of TRPV1^+^ neurons was shown to be sufficient to promote protective IL-17 responses during cutaneous *C. albicans* (and *Staphylococcus aureus*) infection, including eliciting anticipatory type 17 responses in adjacent uninfected skin [277]. The recent demonstration that MrgprD-expressing nonpeptidergic neurons promote cutaneous immune homeostasis and exert immunomodulatory functions during cutaneous *S. aureus* infection raises the possibility of their potential role during skin fungal challenge [278].

As with the oral mucosa, IL-23 produced by DCs and IL-17A produced by CD4^+^ T cells, CD8^+^ T cells, γδ T cells, and ILC3 are critical for protection against cutaneous *C. albicans* infection *in vivo*; instead, IL-22 is dispensable [279]. Three major DC subtypes exist in the skin: Langerhans cells are the only MHCII-expressing cell subset in the epidermis whereas CD11b^+^ DCs and CD103^+^ DCs constitute the dermal DC subsets [280]. Importantly, the morphology of *C. albicans* and the DC subset determine T-helper cell differentiation and fungal control in the skin [281,282]. Thus, yeast cells promote Th17 cell responses—which are critical for cutaneous fungal control— via DECTIN-1-and TLR/MYD88-mediated expression of IL-6 by Langerhans cells in the epidermis, whereas hyphae induce Th1 cell responses—which are dispensable for cutaneous fungal control—but not Th17 cell responses [131,281,283]. Thus, as opposed to Langerhans cells, CD11b^+^ DCs, which also express DECTIN-1, are not required for Th17 cell responses because DECTIN-1 ligation by hyphae does not occur in the dermis. Instead, CD103^+^ DCs, which lack DECTIN-1 expression, suppress Th17 cell development likely through the induction of the inhibitory cytokines IL-12 and IL-27 [282].

Although CD11b^+^ and CD103^+^ DCs are not required for Th17 cell differentiation, they are both important for the production of IL-17A by CD8^+^ T cells in the epidermis, which protects from *C. albicans* skin invasion [284]. Mice deficient in Langerhans cells (or in both Langerhans cells and CD103^+^ dermal DCs) do not exhibit defects in IL-23 production or fungal growth control during cutaneous candidiasis. Instead, CD11b^+^ dermal DCs are both necessary and sufficient for IL-23-mediated, IL-17-driven cutaneous protection against *C. albicans*. Specifically, IL-17-secreting dermal γδ T cells, particularly of the Vγ4 T cell receptor (TCR), constitutively express IL-23R and respond to IL-23 produced by CD301b^+^ dermal DCs to promote *C. albicans* clearance [276].

In a different mouse model of skin fungal abscess formation caused by injection of *C. albicans* hyphae into the deep dermis, a two-step process of initial fungal containment followed by fungal elimination ensues that depends on Nuclear factor of activated T cells (NFAT) signaling, which promotes IL-2 production by DCs and subsequent IFN-γ generation by NK cells. IFN-γ then acts to a) counteract the effects of TGF-β thus limiting myofibroblast differentiation and collagen deposition and to b) promote the generation of plasmin, which mediates collagen capsule digestion, skin ulceration, and elimination of *C. albicans* [285].

*Candida* skin colonization has also been associated with certain skin inflammatory diseases such as atopic dermatitis and psoriasis [286,287]. Recently, cutaneous recall responses to *C. albicans* were shown to promote psoriasiform skin inflammation in mice in a DECTIN-1-, Th17 cell-, neutrophil NET-, and Langerhans cell-dependent manner [288]. These findings support the notion that colonization and/or infection by *C. albicans* may predispose to or amplify psoriasis via the expansion of fungus-reactive Th17 cells.

## Candidemia and systemic candidiasis

In addition to infections at barrier surfaces, *C. albicans*—together with emerging non-*albicans Candida* species—are a leading cause of life-threatening nosocomial bloodstream infections [289–291]. Certain underlying immunosuppressive conditions such as neutropenia and/or corticosteroid administration and medical interventions such as the use of central venous catheters or broad-spectrum antibiotics and chemotherapy- or abdominal surgery-induced gastrointestinal barrier disruption are major risk factors for candidemia and systemic candidiasis, especially in ICU patients [4]. Myeloid phagocytes including neutrophils, inflammatory monocytes, tissue-resident macrophages, and CD11b^+^ DCs are responsible for host defense against systemic candidiasis, whereas T and B lymphocytes and CD103^+^ DCs are dispensable; the only lymphoid cell subset that contributes to systemic anti-*Candida* immunity is innate NK cells [4,109,292–294].

Neutrophils represent the first line of innate defense against systemic candidiasis and neutropenic patients are at heightened risk for development of and suffering from poor outcomes after systemic candidiasis [4,295]. Early neutrophil recruitment and swarming at the site of infection is critical for effective fungal control though the precise host molecular signals that underlie early protective neutrophil responses remain poorly understood [296–299]. The organ-specific ability to rapidly recruit neutrophils correlates with fungal control in mice; thus, the spleen and liver rapidly recruit neutrophils and effectively control *C. albicans*, whereas the kidney exhibits sluggish neutrophil recruitment and is unable to curtail fungal proliferation [40]. During systemic candidiasis, candidalysin contributes to NLRP3 inflammasome activation with subsequent caspase-1-dependent IL-1β secretion and renal neutrophil recruitment [300,301]. In the *C. albicans*-infected CNS, neutrophil recruitment is also facilitated by candidalysin—while Saps are dispensable—which activates CARD9^+^ microglial cells to sequentially produce IL-1β and CXCL1 in a p38- and c-Fos-dependent manner for recruiting protective CXCR2^+^ neutrophils [121,302,303]. Recently, protection from *C. albicans* invasion of the CNS was surprisingly shown to also depend on meningeal IgA-secreting plasma cells that originate from the gut, are positioned adjacent to dural venous sinuses, and facilitate *C. albicans* entrapment in peri-sinus areas to restrict fungal spread in brain tissue [304]; whether meningeal IgA is impaired in CARD9 deficiency remains unknown. Immunization of mice with NDV-3A results in greater CXCL1 levels and improved neutrophil influx into infected tissues leading to decreased fungal burden after systemic *C. albicans* infection [305]. Future clinical studies will be needed to determine whether and how this vaccine may protect humans from systemic candidiasis.

Depending on the size of *C. albicans* structures, recruited neutrophils employ different mechanisms to restrict the fungus [306]. These effector functions include phagocytosis and intracellular killing of *C. albicans* yeast cells via oxidative and non-oxidative cytotoxic mechanisms, degranulation of antimicrobial molecules and formation of NETs to counteract large extracellular fungal hyphae, generation of both pro- and anti-inflammatory cytokines and chemokines, and sequestration of trace elements [307–310]. Mechanisms of NET formation include β-glucan recognition by complement receptor 3 (CR3) in opsonized *C. albicans* whereas for unopsonized *C. albicans*, DECTIN-2 recognition and signaling via SYK and protein kinase delta (PKCδ) result in neutrophil elastase nuclear translocation, histone citrullination, and NETosis, while protein arginine deiminase 4 (PAD4) is dispensable [311–313].

One of the most important antifungal immune effector mechanisms in neutrophils is the generation of ROS via the sequential assembly of the NADPH oxidase complex at the phagosomal membrane and myeloperoxidase (MPO) activation [314]. NADPH oxidase-dependent potassium flux resulting in activation of neutrophil phagosomal proteases is thought to mediate oxidative burst-mediated fungal (including *C. albicans*) killing [315]. In mouse neutrophils, which differ from human neutrophils in their MPO and α-defensin content and activity [316], ROS generation was shown to be dependent on DECTIN-1 recognition leading to calcineurin and NFAT signaling and Mac-1/Vav/PKCδ activation [317]. The importance of ROS in antifungal defense is highlighted by human PIDs that impede ROS production and predispose to systemic fungal infections. For example, chronic granulomatous disease, caused by mutations in 4 out of 5 subunits of the NADPH oxidase complex—with the exception of p40phox—that abrogate oxidative burst, carries a ~40% lifetime risk of pulmonary aspergillosis [318,319], whereas systemic *Candida* infections occur less frequently (<5-10%) and often involve atypical anatomical niches such as the lymph nodes. Similarly, humans with complete MPO deficiency infrequently (~5%) suffer from systemic candidiasis, typically in the presence of additional predisposing factors such as diabetes mellitus [320]. Collectively, these observations highlight the critical contribution of compensatory non-oxidative mechanisms in *Candida* clearance.

Neutrophil non-oxidative fungal killing mechanisms include AMPs, hydrolases, and nutritional immunity. Two recently recognized molecular signals that mediate neutrophil granulogenesis, degranulation, and non-oxidative *C. albicans* killing include the endoplasmic reticulum transmembrane protein Jagunal homolog 1 (JAGN1) and the chemokine receptor CXCR1 [321,322]. In fact, the mutant CXCR1 allele *CXCR1-T276* was shown to impair neutrophil degranulation and *C. albicans* killing and was associated with an increased risk of disseminated candidiasis in infected patients [321]. Moreover, using neutrophils from patients with various PIDs two independent signaling mechanisms were characterized that control phagolysosomal function and oxidative or non-oxidative burst-dependent killing in response to opsonized and unopsonized *C. albicans* yeast cells [323]. Specifically, killing of opsonized *C. albicans* occurs in a DECTIN-1-independent and SYK-dependent manner and relies on the NADPH oxidase system, Fcγ receptors, and protein kinase C (PKC). By contrast, killing of unopsonized *C. albicans* yeast cells by human neutrophils occurs independently of the NADPH oxidase system and relies on CR3, CARD9, and phosphoinositide-3-kinase (PI3K) [323].

Although crucial for fungal control, neutrophil-mediated immunity may also come at the cost of immunopathology and tissue injury, particularly in the renal tubules within which neutrophils and *C. albicans* invade [324]. The molecular mediators that underlie pathogenic neutrophil effects have been uncovered in the mouse model of systemic candidiasis. For example, excessive neutrophil recruitment during the late phase of infection is CCR1-dependent exerting detrimental effects on renal function and host survival [296,299,325]. Moreover, leukotriene B4-dependent neutrophil accumulation in the *C. albicans*-infected lung results in pulmonary capillaritis and hemorrhage and hypoxia [326]. Furthermore, the tyrosine kinase Tec, the suppressor of TCR signaling (Sts) phosphatases, the lectin galectin-3, the endoribonuclease MCPIP1, and IL-17C are also implicated in neutrophil-mediated immunopathology in *C. albicans*-infected tissues [327–330]. By contrast, DCs expressing dendritic cell natural killer lectin group receptor-1 (DNGR-1) inhibit renal CXCL2 expression and decrease neutrophil recruitment to ameliorate neutrophil-induced tissue damage during systemic candidiasis [331]. In addition, IL-17R signaling on renal tubular epithelial cells (RTECs) activates the Kallikrein-kinin system and protects RTEC from caspase-3-dependent apoptosis and ameliorates renal damage following systemic candidiasis [332]. Thus, although neutrophils play a critical role in defense against systemic candidiasis, their prolonged and/or excessive recruitment and activation may exert damaging effects. Additional studies are needed to delineate the complex tissue-specific regulatory networks that control the spatial and temporal regulation of neutrophil accumulation and function and to define the relevance of these pathways in humans with systemic candidiasis, in whom neutrophil-associated immunopathology has been observed in the settings of hepatosplenic candidiasis during neutrophil recovery and of renal candidiasis [333–335].

Besides neutrophils, mononuclear phagocytes also promote protective host defense during systemic candidiasis [293]. Specifically, inflammatory Ly6C^hi^ monocytes, which migrate in infected tissues and differentiate into macrophages and monocyte-derived DCs, as well as tissue-resident macrophages, and DCs contribute to fungal clearance through both direct anti-*Candida* effector functions such as phagocytosis, fungal killing, cytokine production, antigen presentation, and inflammasome activation, and via boosting ROS generation and/or the candidacidal activity of neutrophils [112,336,337]. Inflammatory monocytes traffic into the *C. albicans*-infected kidney and CNS in a CCR2-dependent manner, can directly inhibit *C. albicans* growth, and are critical for fungal clearance in these tissues and host survival [338]. Inflammatory monocytes also promote the candidacidal activity of neutrophils. Specifically, splenic inflammatory monocytes produce IL-15 in a type I interferon-dependent manner and activate CCR5-recruited NK cells to produce GM-CSF, which in turn boosts the *Candida* killing capacity of renal neutrophils [337,339]; this NK function was shown to rely on IL-17R signaling [340]. Besides inflammatory monocytes, CD11b^+^ DCs depend on SYK signaling to generate IL-23, which represents another local renal molecular mechanism for augmenting GM-CSF production by NK cells and enhancing neutrophil candidacidal activity [112]. IL-23 also provides survival signals to neutrophils within the *C. albicans*-infected kidney acting in a partially autocrine, IL-17-independent manner to inhibit apoptosis and protect from infection [341]. Last, CD169^+^ renal macrophages represent another tissue-resident mononuclear phagocyte subset that contributes to priming neutrophil ROS production via IFN-γ and control of *C. albicans* renal proliferation [292].

In addition, renal tissue-resident macrophages form direct contacts with *C. albicans* yeast and hyphal forms within the first few hours following infection and exhibit candidacidal activity [324]. The chemokine receptor CX3CR1 is fundamental for control of *C. albicans* growth in the kidney and host survival by promoting renal macrophage accumulation, direct macrophage-*C. albicans* interactions, and macrophage killing. Mechanistically, CX3CR1 modulates macrophage survival by inhibiting caspase-3-dependent apoptosis associated with AKT activation [324]. In humans, the dysfunctional *CX3CR1-M280* allele was associated with increased risk for developing candidemia and poor outcome after infection [324]. Mechanistically, individuals homozygous for the *CX3CR1-M280* allele were shown to exhibit a defect in CX3CL1-mediated monocyte survival due to impaired AKT and ERK activation and had low blood monocyte counts at the steady state [342]. By contrast, CX3CR1-expressing macrophages are dispensable for OPC and VVC control in mice and humans [343]. However, in the gut, CX3CR1-expressing mononuclear phagocytes modulate the composition of and respond to gut fungal communities in a CLR/SYK-dependent manner and patients with IBD carrying the *CX3CR1-M280* polymorphism have reduced antifungal antibody responses [344]. CX3CR1-expressing gut macrophages also respond to gut *C. albicans* to promote the expansion of germinal center-dependent B lymphocytes for the development of antifungal IgG responses that protect from systemic fungal challenge; this response is abrogated in the setting of CARD9 deficiency [20].

Upon encountering *C. albicans*, macrophages up-regulate signaling pathways involved in phagocytosis and inflammation [345]. The tetraspanin CD82 promotes clustering of DECTIN-1 in the phagocytic cup and DECTIN-1-dependent SYK signaling and mediates macrophage fungal killing and pro-inflammatory cytokine responses. Accordingly, *Cd82*^−/−^ mice fail to control fungal growth and exhibit greater susceptibility *in vivo* and polymorphisms in the *CD82* gene are associated with development of candidemia in patients [346]. To facilitate engulfment of long hyphal filaments, macrophages can fold fungal hyphae in a process that involves hyphal sensing by DECTIN-1 and β2-integrin and polymerization of the actin–myosin filaments of the phagosome [347]. To avoid rupture of the phagosome and maintain its integrity, macrophages increase the phagosome surface area by lysosome biosynthesis and fusion which is modulated by the transcriptional regulator TFEB [348]. *C. albicans*-mediated neutralization of the phagosome and yeast-to-hyphal transition trigger NLRP3-dependent lytic pyroptosis in macrophages [349–351]. CLR/SYK-mediated negative regulation of macrophage function during systemic candidiasis also occurs. Specifically, the E3-ubiquitin ligase CBLB targets DECTIN-1, DECTIN-2, and SYK for ubiquitination and degradation in macrophages (and DCs) and leads to impaired inflammasome activation, oxidative burst, and fungal killing and increased mortality during systemic candidiasis [352,353]. In addition, down-regulation of the CLR FcεRII (CD23) by engaging JNK1 signaling downstream of DECTIN-1 ligation in macrophages (and DCs) compromises FcεRII-mediated nitric oxide production and increases mortality during systemic candidiasis [354]. Thus, targeting CBLB and FcεRII may have therapeutic implications for systemic candidiasis.

Furthermore, recent studies have underscored the importance of modulating host metabolism in influencing phagocyte-mediated responses to systemic *C. albicans* infection. For example, CLR-mediated metabolic reprogramming of monocytes and macrophages mainly via induction of glucose metabolism and increased glycolysis is important for protection against systemic candidiasis [355]. To avoid clearance, *C. albicans* perturbs host glucose homeostasis by depleting glucose and triggering rapid macrophage death, which depend on glycolysis for energy [356]. Moreover, glutathione reductase (Gsr)-mediated redox regulation is necessary for *C. albicans* clearance by neutrophils and macrophages. *Gsr*^−/−^ mice had increased kidney fungal burden, enhanced cytokine and chemokine responses, greater neutrophil infiltration in the infected kidney and heart, and increased mortality during systemic candidiasis. Mechanistically, Gsr deficiency led to defective phagocytosis, respiratory burst, and fungicidal activity in neutrophils and increased levels of pro-inflammatory cytokines and MAPK and SYK activities in macrophages [357]. In addition, restoration of glucose uptake in neutrophils by pharmacological inhibition of glycogen synthase kinase 3 beta (GSK3β) rescued ROS production and candidacidal function of neutrophils from uremic mice and patients with chronic kidney disease [358].

The ability of cells to exhibit immunological memory was thought of as an exclusive feature of the adaptive immune system, however activation of monocytes and macrophages can also result in enhanced responsiveness to subsequent triggers via a process termed trained immunity, which is mediated by epigenetic reprogramming [359]. First described in the setting of *C. albicans* infection in 2012, trained immunity promotes T and B lymphocyte-independent protection against systemic candidiasis following a first exposure to a non-lethal infectious dose. This protection is conferred by monocytes and macrophages via DECTIN-1/Raf-1/NF-κB activation after exposure to β-glucan [360,361]. Trained monocytes and macrophages exert enhanced pro-inflammatory responses and exhibit a metabolic shift toward aerobic glycolysis via AKT/mTOR/HIF-1α signaling [361].

In summary, the delineation of the molecular basis of antifungal host defense mechanisms against life-threatening systemic candidiasis holds promise for the identification of genetic variants in immune-related genes, which—either alone or in combination—may explain patient-specific susceptibility to infection. Besides the aforementioned genetic variation in the *CXCR1, CX3CR1, CD82,* and TLR gene loci [145,321,324,346], additional population studies have revealed increased susceptibility to systemic candidiasis in patients with certain genetic variants in the *TNF, IL10, IL12B, CCL8, CD58, TAGAP, LCE4A-C1orf68, PSMB8, SP110*, and *STAT1* genes; strikingly, the combinatorial presence of certain genetic variants has been reported to lead to up to ~20-fold increases in host susceptibility to candidemia [144,362–365]. Collectively, these findings may eventually help devise personalized immunogenetics-based strategies that could allow for risk stratification, intensified diagnosis, targeted vaccination, antifungal prophylaxis, and/or prognostication of patients in the ICU, which may improve their outcomes.

## Therapeutic interventions and drug resistance mechanisms

**Figure 3.**
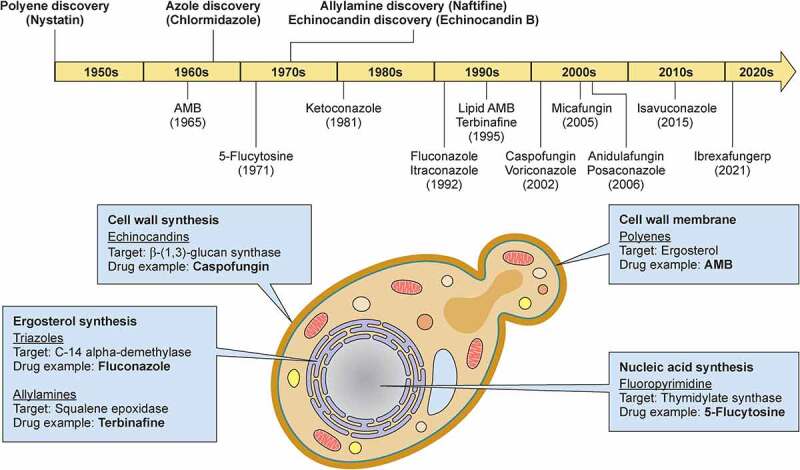
Milestones in antifungal drug development and fungal targets of the currently available antifungal agents. In the upper panel, the timeline depicts the date of discovery of the first indicated antifungal compound within each class of antifungal drugs and the date of FDA approval for the most common antifungal drugs with anti-*Candida* activity. In the lower panel, a *C. albicans* budding yeast is depicted and the targets of antifungal drugs are shown. Illustration created with BioRender.com. AMB, amphotericin B.

Factors contributing to the high morbidity and mortality of systemic candidiasis in patients include the poor performance of fungal diagnostics (reviewed elsewhere [366]) and the suboptimal efficacy of antifungal drugs *in vivo*. Currently available classes of antifungal drugs that are used to treat *C. albicans* infections include the polyenes, 5-flucytosine (5-FC), the azoles, and the echinocandins [367]. Terbinafine is an allylamine antifungal drug that inhibits ergosterol biosynthesis by targeting squalene epoxidase and blocking the conversion of squalene to squalene epoxide [368]. However, its use is restricted to the treatment of onychomycosis and cutaneous fungal infections due to its limited systemic bioavailability [369], and thus will not be further discussed here (Figure 3).

The oldest class of antifungal drugs available in the clinic are polyenes. Polyenes bind to ergosterol, a cell membrane sterol that is unique to fungi. Upon binding, polyenes form pores in the fungal cell membrane causing osmotic cell lysis. Besides pore formation, the mode of action of amphotericin B (AMB) also involves oxidative damage to fungal cells [370] and ergosterol sequestration, leading to extra-membranous aggregates [371]. The most recognizable member of the polyene family is AMB, which remains one of the most potent and broad-spectrum antifungal agents for the treatment of mucosal and systemic fungal infections, whereas nystatin, another polyene, is solely used topically for the treatment of OPC [372,373]. Nephrotoxicity is a common and limiting adverse effect to AMB, although the lipid drug formulations cause less renal damage [374]. The recent advent of the cochleated formulation of AMB shows promise for efficacious oral drug delivery without its nephrotoxic effects [375]. Despite the widespread use of AMB over decades, resistance of *C. albicans* to AMB is very uncommon and, when present, it is associated with mutations in the *ERG3* or *ERG6* genes of the ergosterol biosynthesis pathway, which result in decreased levels of ergosterol thus impeding the binding of AMB to the fungal cell membrane [376,377].

5-FC is a pyrimidine analog that is taken up by *C. albicans* and converted to 5-fluorouracil, which interferes with fungal DNA and RNA synthesis. The rapid development of *C. albicans* resistance to 5-FC when used as monotherapy, caused by mutations in cytosine permease and cytosine deaminase that decrease uptake or conversion of the drug to 5-fluorouracil, respectively, and the hematological, hepatic, and gastrointestinal adverse effects of the drug limit its use in the clinic [378]. Thus, 5-FC is typically administered in combination with AMB or triazoles in the setting of certain difficult-to-treat infections such as candidal endocarditis or meningitis [379].

The azoles target Erg11p/Cyp51 and inhibit the biosynthesis of ergosterol leading to the accumulation of toxic sterols on the fungal cell membranes such as 14α-methylergosta-8,24(28) dienol and increased levels of endogenous ROS [380,381], both of which contribute to fungal growth arrest. Azoles are divided into two subgroups based on their chemical structure: the imidazoles such as clotrimazole, ketoconazole, and miconazole, and the triazoles, which include fluconazole, itraconazole, voriconazole, posaconazole, and isavuconazole. The topical application of imidazoles is used for the treatment of mucosal candidiasis, whereas the triazoles are commonly used to treat mucosal and systemic infections by *C. albicans*, although triazole-specific toxicities and drug-drug interactions occasionally restrict their clinical use [382]. The emergence of azole resistance poses therapeutic challenges [383]. It is observed more often during treatment of patients with CMC compared to those with candidemia due to the recurrent nature of these infections and the repeated exposures to azoles [384–386]. The molecular mechanisms of azole resistance in *C. albicans* include: mutations in the *ERG11* gene that result in *ERG11* overexpression; gain-of-function mutations in the ergosterol biosynthesis pathway regulator *UPC2* gene that also lead to increased *ERG11* expression; mutations in the *ERG3* or *ERG6* genes that cause accumulation of toxic sterols; overexpression of drug efflux pumps including the ABC transporters *CDR1* and *CDR2* and the MFS transporter *MDR1* caused by gain-of-function mutations in the transcription factors *TAC1* and *MRR1*; genomic plasticity in the forms of aneuploidy, trisomy, loss of heterozygosity, and isochromosome formation; as well as enzymatic changes involved in the sphingolipid synthesis pathway [387,388]. Addition of a tetrazole group by substitution of the triazole metal-binding group has resulted in decreased drug-drug interactions and improved tolerability due to the greater specificity for the fungal *ERG11* over the human *CYP51* [389]. The tetrazole compounds VT-1161/oteseconazole and VT-1598 exhibit superior *in vitro* and *in vivo* activity against clinical *C. albicans* (including azole-resistant) strains from patients with CMC [390,391], and oteseconazole was safe and efficacious for the treatment of recurrent VVC in a Phase II randomized, double-blind, placebo-controlled clinical trial [392].

The most recently introduced class of antifungal drugs in the clinic, the parenterally used echinocandins, consist of the well-tolerated caspofungin, micafungin, and anidulafungin, which inhibit β-(1,3)-glucan synthase, an enzyme involved in the generation of β-(1,3)-glucan; in the absence of β-(1,3)-glucan, loss of fungal cell wall rigidity and cell lysis ensue [393]. The increasing incidence of echinocandin-resistant *C. albicans* clinical strains in recent years is concerning [394]. The most common mechanism of resistance relates to mutations in hotspot regions of the β-(1,3)-glucan synthase gene *FKS* [395], and are typically associated with prior echinocandin exposure [396,397]. In addition, although more prevalent in *C. glabrata* [398], mutations in the mismatch repair gene *MSH2* can also result in echinocandin resistance in *C. albicans; MSH2* mutations often confer cross-resistance to azoles and, alarmingly, may occur without prior exposure to echinocandins [399]. Recently, ibrexafungerp, an oral antifungal drug that belongs to a novel class of glucan synthase inhibitors termed triterpenoids, has shown significant activity against *C. albicans* (and multidrug-resistant *C. glabrata and C. auris*). Ibrexafungerp maintains its efficacy at low pH conditions which are often encountered in the vaginal mucosa and was recently FDA-approved for the treatment of VVC in women [400,401].

Another promising antifungal drug currently in Phase II clinical trials is the first-in-class fosmanogepix, an inhibitor of the fungal enzyme Gwt1, which is involved in glycosylphosphatidylinositol-anchored mannoprotein biosynthesis, trafficking, and anchoring to the cell membrane and outer cell wall [402]. Fosmanogepix exhibits a broad spectrum *in vitro* activity against *C. albicans* (including echinocandin-resistant) strains, other yeast, and mold fungi and has shown significant *in vivo* efficacy in mouse and rabbit models of OPC and disseminated *C. albicans* infections [403–405]. Although developing antifungal drugs is hindered by the evolutionary proximity between eukaryotic fungi and humans, additional novel antifungal agents are in different phases of development and promising candidates such as turbinmicin have recently emerged via metabolomic screens from the microbiome of marine animals [406–408]. For a more detailed review of the new antifungal agents in clinical development the reader is refered to [409].

## Outlook

The co-evolution of *C. albicans* with humans has established a complex balance between commensalism and pathogenicity for this yeast fungus. In recent decades, advances in modern medicine have enabled this pathobiont to become one of the most common human pathogens causing life-threatening healthcare-associated infections, the prevalence of which continues to be significant. Understanding the virulence traits of *C. albicans*, the tissue-specific mechanisms of anti-*Candida* host defense, and its mechanisms of resistance to the armamentarium of available antifungal drugs should enable the development of better strategies for the diagnosis and treatment of affected individuals, which may help improve patient outcomes.

## Data Availability

No data sets were analyzed in this manuscript, and data availability is not applicable.

## Abbreviations

Vulvovaginal candidiasis (VVC), Oropharyngeal candidiasis (OPC), Esophageal candidiasis (EPC), Central nervous system (CNS), Antimicrobial peptides (AMPs), Platelet-derived growth factor BB (PDGF BB), Neural precursor cell expressed developmentally down-regulated protein 9 (NEDD9), Epidermal growth factor receptor (EGFR), Secreted aspartic proteinases (Saps), N-acetylglucosamine (GlcNAc), Superoxide dismutases (Sods), Reactive oxygen species (ROS), Heat shock proteins (Hsps), Pathogen-associated molecular patterns (PAMPs), Pattern recognition receptors (PRR), MAPK (mitogen-activated protein kinase), CEK1 (Choline/ethanolamine kinase 1), C-type lectin receptors (CLRs), Spleen tyrosine kinase (SYK), Caspase recruitment domain-containing protein 9 (CARD9), Nuclear factor kappa-light-chain-enhancer of activated B cells (NF-κB), NF-κB-inducing kinase (NIK), Neutrophil extracellular traps (NETs), Primary immunodeficiency disorder (PID), Recurrent VVC (RVVC), Toll-like receptors (TLRs), Intensive care unit (ICU), NOD-like receptors (NLRs), Nicotinamide adenine dinucleotide phosphate (NADPH), Chronic mucocutaneous candidiasis (CMC), monoclonal antibodies (mAbs), Inflammatory bowel disease (IBD), type 3 innate lymphoid cells (ILC3), Dendritic cells (DCs), natural Th17 (nTh17), Tissue-resident memory T cells (T_RM_), Granulocyte colony-stimulating factor (G-CSF), Granulocyte-macrophage colony-stimulating factor (GM-CSF), Autoimmune polyendocrinopathy–candidiasis–ectodermal dystrophy (APECED), Autoimmune polyglandular syndrome type 1 (APS-1), Autoimmune regulator (AIRE), Interferon gamma (IFN-γ), IL-1 receptor antagonist (IL-1Ra), Phosphoglycerate mutase (Gpm1), Calcitonin gene-related peptide (CGRP), T cell receptor (TCR), Complement receptor 3 (CR3), PKCδ (protein kinase delta), NFAT (Nuclear factor of activated T cells), Protein arginine deiminase 4 (PAD4), Myeloperoxidase enzyme (MPO), Jagunal homolog 1 (JAGN1), Protein kinase C (PKC), Phosphoinositide-3-kinase (PI3K), Suppressor of TCR signaling (Sts), Dendritic cell natural killer lectin group receptor-1 (DNGR-1), Renal tubular epithelial cells (RTECs), Glutathione reductase (Gsr), Glycogen synthase kinase 3 beta (GSK3β), 5-flucytosine (5-FC), Amphotericin B (AMB).
